# Potential use of antioxidants for the treatment of chronic inflammatory diseases

**DOI:** 10.3389/fphar.2024.1378335

**Published:** 2024-05-16

**Authors:** Alexander V. Blagov, Volha I. Summerhill, Vasily N. Sukhorukov, Elena B. Zhigmitova, Anton Y. Postnov, Alexander N. Orekhov

**Affiliations:** ^1^ Institute of General Pathology and Pathophysiology, Moscow, Russia; ^2^ Institute for Atherosclerosis Research, Moscow, Russia; ^3^ Laboratory of Cellular and Molecular Pathology of Cardiovascular System, Federal State Budgetary Scientific Institution, Petrovsky National Research Centre of Surgery (FSBSI “Petrovsky NRCS”), Moscow, Russia

**Keywords:** ROS, chronic inflammation, antioxidants, chronic inflammatory diseases, cellular antioxidant enzymes

## Abstract

The excessive production of various reactive oxidant species over endogenous antioxidant defense mechanisms leads to the development of a state of oxidative stress, with serious biological consequences. The consequences of oxidative stress depend on the balance between the generation of reactive oxidant species and the antioxidant defense and include oxidative damage of biomolecules, disruption of signal transduction, mutation, and cell apoptosis. Accumulating evidence suggests that oxidative stress is involved in the physiopathology of various debilitating illnesses associated with chronic inflammation, including cardiovascular diseases, diabetes, cancer, or neurodegenerative processes, that need continuous pharmacological treatment. Oxidative stress and chronic inflammation are tightly linked pathophysiological processes, one of which can be simply promoted by another. Although, many antioxidant trials have been unsuccessful (some of the trials showed either no effect or even harmful effects) in human patients as a preventive or curative measure, targeting oxidative stress remains an interesting therapeutic approach for the development of new agents to design novel anti-inflammatory drugs with a reliable safety profile. In this regard, several natural antioxidant compounds were explored as potential therapeutic options for the treatment of chronic inflammatory diseases. Several metalloenzymes, such as superoxide dismutase, catalase, and glutathione peroxidase, are among the essential enzymes that maintain the low nanomolar physiological concentrations of superoxide (O_2_•−) and hydrogen peroxide (H_2_O_2_), the major redox signaling molecules, and thus play important roles in the alteration of the redox homeostasis. These enzymes have become a striking source of motivation to design catalytic drugs to enhance the action of these enzymes under pathological conditions related to chronic inflammation. This review is focused on several major representatives of natural and synthetic antioxidants as potential drug candidates for the treatment of chronic inflammatory diseases.

## 1 Introduction

Inflammation is a pathophysiological process characterized by the accumulation of inflammatory cells, cytokines, and chemokines. According to the duration of inflammation process, there are acute and chronic stages. A sudden onset, a short duration, the presence of exudative lesions, and granulocyte infiltration are features of the acute inflammation. On the contrary, chronic inflammation can last months to years and is often governed by hyperplasia lesions with macrophage and lymphocyte infiltration. Upon infection or trauma, acute inflammation is induced, becoming the central mechanism, by which the innate immune system eliminates invading pathogens (Chakraborty and Lo, 2020). However, excessive stimulation of immune system may result in cytokine storms, sepsis followed by multiple organ dysfunction, the major cause of hospital deaths (Rostamtabar et al., 2021). If the tissue damage or infection persist, aggregation of inflammatory cells and cytokines can transform acute inflammation into the chronic stage, potentially generating local and systemic detrimental effects (Feehan and Gilroy, 2019). Interestingly, chronic inflammation can be characterized by a formation of vicious inflammatory cycle, when the inflammatory process can maintain itself, exacerbating the course of disease (Tschopp, 2011).

Numerous human diseases, including age-related neurodegenerative disorders such as Alzheimer’s disease and Parkinson’s disease, are associated with chronic inflammation. The most common chronic inflammatory diseases are atherosclerosis, cardiovascular diseases (CVDs), rheumatoid arthritis, psoriasis, type 2 diabetes, Crohn’s disease, ulcerative colitis, multiple sclerosis, and chronic obstructive pulmonary disease, among others. Moreover, the interplay between inflammation and cancer development is a well-known phenomenon (Khandia and Munjal, 2020). Increasing evidence suggests that oxidative stress is involved in the physiopathology of various diseases associated with chronic inflammation that require continuous pharmacological treatment (García-Sánchez et al., 2020). In recent years, a great deal of research has been dedicated to free radical chemistry (Tvrdá and Benko, 2020) and there are undisputable pieces of evidence indicating that free radicals can cause the oxidative damage of biomolecules such as proteins, lipids, carbohydrates, and nucleic acids and thus alter the intracellular redox state, leading to the development of oxidative stress (Engwa et al., 2022). Thus, the control of oxidative stress processes may become fundamental in both the prevention and treatment of chronic inflammatory diseases.

The number of patients suffering from chronic inflammatory diseases has increased dramatically over the past 3 decades (Netea et al., 2017). More than 50% of deaths in the world are due to diseases associated with inflammation, which makes it a priority concern for modern healthcare (Furman et al., 2019). The situation is aggravated by high financial costs for medical care. In the United States alone, chronic inflammatory diseases and related chronic syndromes encounter a broad-range of costs depending on the severity of disease, number of high-cost events, therapies applied, and treatment compliance rates (Wylezinski et al., 2019). In Europe, the cost of medical care for patients suffering from inflammatory bowel diseases, for example, is estimated at 4.6–5.6 billion euros per year (Burisch et al., 2013). The pharmacotherapy, including nonsteroidal anti-inflammatory drugs, glucocorticoids, and monoclonal antibodies remains a standard option for the treatment of chronic inflammatory diseases, although these drugs can have severe side effects and are not always effective enough (Bindu et al., 2020; Oray et al., 2016; Ordás et al., 2012). The shortcomings of existing medicines, the growing incidence of inflammatory disorders, and high financial costs associated with their treatment created the need to develop new therapeutic approaches for the treatment of these disorders. Therefore, this review is focused on several major representatives of natural and synthetic antioxidants as potential drug candidates for the treatment of chronic inflammatory diseases.

## 2 Involvement of ROS in cellular apoptosis and chronic inflammation

ROS are generally defined as partially reduced oxygen metabolites with powerful oxidizing capabilities that play important roles in cell signaling and pathophysiology of inflammatory disorders. The widely-studied ROS include superoxide anion (O_2_•−), hydroxyl anions (OH^−^), hydroxyl radical (•OH), hydrogen peroxide (H_2_O_2_), and hypochlorous acid (HOCl), the properties of which have been described elsewhere (Sies and Jones, 2020; Sun et al., 2020). Although, there are other ROS, such as organic hydroperoxides (ROOH), peroxyl radical (ROO•), alkoxyl radical (RO•), singlet molecular oxygen (^1^O_2_), and ozone (O_3_), that also play essential roles in signaling and disease (Sies and Jones, 2020).

It is well-known that mitochondria are the major source of ROS, as well as their main targets in eukaryotic cells. Superoxide anions are the most abundant ROS in the mitochondria (Zorov et al., 2014). Interestingly, only 0.2%–2.0% of the molecular oxygen taken by mitochondria is reduced to O_2_•−, which subsequently is converted to H_2_O_2_, which represents the major oxidative load (Balaban et al., 2005; Zhao et al., 2019). Other researches also reported that mitochondria are, by quantitative definition, the most important sources of O_2_•− and H_2_O_2_ in mammalian cells (Mailloux, 2020). In mitochondria, ROS are produced as toxic by-products of metabolism via oxidative phosphorylation, i.e., electron leakage from the electron transport chain (predominantly from complexes I and III), or via reactions involving cytochrome P450 (Mittal et al., 2014). Complexes I and III of the respiratory chain of the mitochondria have been recognized as ROS sources (Nickel et al., 2014). Additional sources of mitochondrial ROS are pyruvate dehydrogenase, 2-oxoglutarate dehydrogenase, dihydroorotate dehydrogenase, and p66shc/cytochrome *c* (Lambert and Brand, 2009; Mailloux et al., 2013). Increased production of mitochondrial ROS plays a key role in the development of inflammatory responses (Bulua et al., 2011), leading to a dysfunction in cell components and organelles, especially mitochondria. In turn, mitochondrial dysfunction can lead to overproduction of ROS, which can facilitate oxidative damage of mitochondrial DNA. The brains of people with Alzheimer’s disease show high levels of oxidative damage to mtDNA, approximately 10 times greater than in people without the disease (Cioffi et al., 2019). Mitochondrial DNA appears to be particularly sensitive to oxidative damage, and thus prone to mutations. Increased sensitivity to mutations of mitochondrial DNA is due to its close proximity to the site of ROS production and the lack of protective histones. Aged individuals, whose cells have accumulated a high level of oxidative damage, show an increased rate of mutagenesis in their mitochondrial DNA (Kujoth et al., 2005). There are cellular sources of ROS, such as reactions involving xanthine oxidoreductase, the enzyme that catalyzes the oxidation of hypoxanthine to xanthine and uric acid (Liu et al., 2021).

Another source of non-specific ROS formation is reactions involving the NO-synthase (NOS), which catalyzes the production of nitric oxide and citrulline from arginine. NOS isoforms catalyze the production of superoxide through the enzyme nicotinamide adenine dinucleotide phosphate (NADPH) oxidase (NOX family enzymes). The NOX family of proteins is located in the plasma, endoplasmic reticulum, and mitochondrial membranes. NOX catalytic subunits transfer electrons from NADPH through cell membranes to molecular oxygen to produce O_2_•−. In this way, NADPH oxidases catalyze the reduction of molecular oxygen to O_2_•− (Chandel, 2021). Moreover, NOX enzymes, such as NOX4, DUOX1, and DUOX2, are prominent sources of H_2_O_2_ linked to aberrant cellular signaling, through which the development of cancer and metastasis may be promoted (Meitzler et al., 2019). All NOX enzymes encompass six transmembrane domains with conserved flavin adenine dinucleotide and NADPH binding sites and four heme-binding histidines in the third and fifth transmembrane domains to generate ROS by donating an electron to molecular oxygen (Drummond and Sobey, 2014). Each member of the NOX family holds the catalytic domain permitting the electron transfer from cytosolic NADPH during the generation of ROS metabolites. Such transfer leads to the reduction of oxygen by NOX/DUOX to O_2_•− and the production of secondary ROS by-products (Bedard and Krause, 2007). In this process, two O_2_•− molecules have the potential to react generating H_2_O_2_, either naturally, or under the influence of SOD during the dismutation reaction. H_2_O_2_ can further react with O_2_•− in the presence of iron, producing •OH. The NADPH oxidases are present in various cells, particularly the professional phagocytes and endothelial cells (Vermot et al., 2021), which play an important role in the generation of the inflammatory response. Their activation occurs through different pathways including phosphorylation of cytosolic regulatory subunits by protein kinase C, protein kinase A, phosphatidylinositol-3-kinase (PI3K), mitogen-activated protein kinases (MAPK), and non-receptor associated protein kinases, such as Janus kinase (JAK) and Src, the non-receptor tyrosine kinases (Manea et al., 2015).

ROS can directly cause damage to biological macromolecules, such as proteins, lipids, and nucleic acids, and that eventually leads to cell death (Auten and Davis, 2009). In particular, the oxidation of proteins leads to a disruption of the whole complex of enzymatic cascades, resulting in cellular dysfunction and subsequent cell death (Stadtman and Levine, 2000). Besides, ROS are involved in the process of lipid peroxidation (Fruhwirth and Hermetter, 2008). This review presented the evidence indicating that during the oxidation of phospholipids, which are the main components of the cytoplasmic membrane, the enzyme sphingomyelinase is activated followed by the formation of ceramide from sphingomyelin. Ceramide is not only a component of the cytoplasmic membrane but also a signaling molecule involved in the activation of apoptosis. The oxidation of DNA is associated with the formation of DNA chain breaks, which leads to necrosis and/or maladaptive apoptosis of the cell (Auten et al., 2002). In addition, superoxide anions combined with nitric oxide yield peroxynitrite (ONOO-), a strong oxidant capable of affecting the integrity of mitochondria, as reviewed in Pérez de la Lastra et al. (2022). In that regards, the term oxidative stress was defined as an imbalance between oxidants and antioxidants in favor of the oxidants, resulting in molecular damage and/or disruption of redox signaling and control (Sies, 2020). However, the detrimental effects of ROS on cells are manifested only at sufficiently high concentrations, while at low or “physiological” concentrations (exact concentrations remain to be verified), ROS promote complex signaling functions, including the regulation of cell growth, differentiation, adhesion, initiation of apoptosis, and senescence, as well as antimicrobial effects (Thannickal and Fanburg, 2000). For example, H_2_O_2_ is a central redox signaling molecule in redox metabolism and its amount is vital to oxidative stress. Under physiological conditions, when H_2_O_2_ intracellular concentration is 1–10 nM, it regulates the stress response implicated in the physiological and adaptive processes termed oxidative eustress. Whereas, higher concentrations (over 100 nM) are accountable for the so-called oxidative distress, in which the induced inflammatory response leads to cell damage (Sies, 2017). The review of Sies and Jones (2020) described many signaling mechanisms involved in cell survival and tissue function that require certain levels of ROS (Sies and Jones, 2020). A key mechanism by which ROS mediate their biological effects in redox regulation is via thiol-based modification of target proteins (Zeida et al., 2019). Apart from participating in normal physiological cellular processes, ROS are also known for their role in mediating pathophysiological signal transduction. ROS-associated apoptosis can take place by an external or internal mechanisms. The external pathway is initiated by proinflammatory macrophages that secrete inflammatory cytokines in addition to ROS. These signaling molecules bind to the so-called death receptors on the membrane of target cells, which triggers a chain of cell death reactions, leading to tissue damage in the site of inflammation (Mittal et al., 2014). The main receptors of cell death include the receptors of the following ligands: tumor necrosis factor alpha (TNFα) (TNFR1), TRAIL1 (TRAIL-R1), TRAIL2 (TRAIL-R2), and the Fas (Circu and Aw, 2010). Subsequent reactions include activation of proteins of the death domains, such as FADD and TRADD, which in turn activate procaspase-8 with the formation of caspase-8, which triggers the effector protein caspase-3, that plays one of the key roles in the apoptotic process (Circu and Aw, 2010). The internal mechanism is activated in the mitochondrial cell, in which the increased levels of ROS cause damage and increased permeability of the mitochondrial membrane. The action of apoptotic Bax/Bak proteins also contribute to the mitochondrial membrane permeability increase. That leads to cytochrome c entry into the cytoplasm, where it acts as a potent apoptosis inducer, forming the apoptosomal complex with the apoptotic peptidase activating factor 1 (Apaf-1) protein, into which procaspase-9 is embedded and activated to caspase-9, which in turn activates effector caspase-3 that plays a central role in the execution-phase of cell apoptosis (Green and Kroemer, 2004). In this way, mitochondria, the major source of intracellular ROS, are crucial regulators of the intrinsic apoptotic pathway. Damage to the mitochondrial components, primarily the elements of the electron transport chain, disrupts the functioning of the mitochondria and adenosine triphosphate (ATP) production and the entire cell as a result of energy metabolism disruption. Mitochondrial dysfunction was found to be related to a number of acute and chronic inflammatory pathologies and the process of aging (reviewed in López-Armada et al., 2013).

**FIGURE 1:**
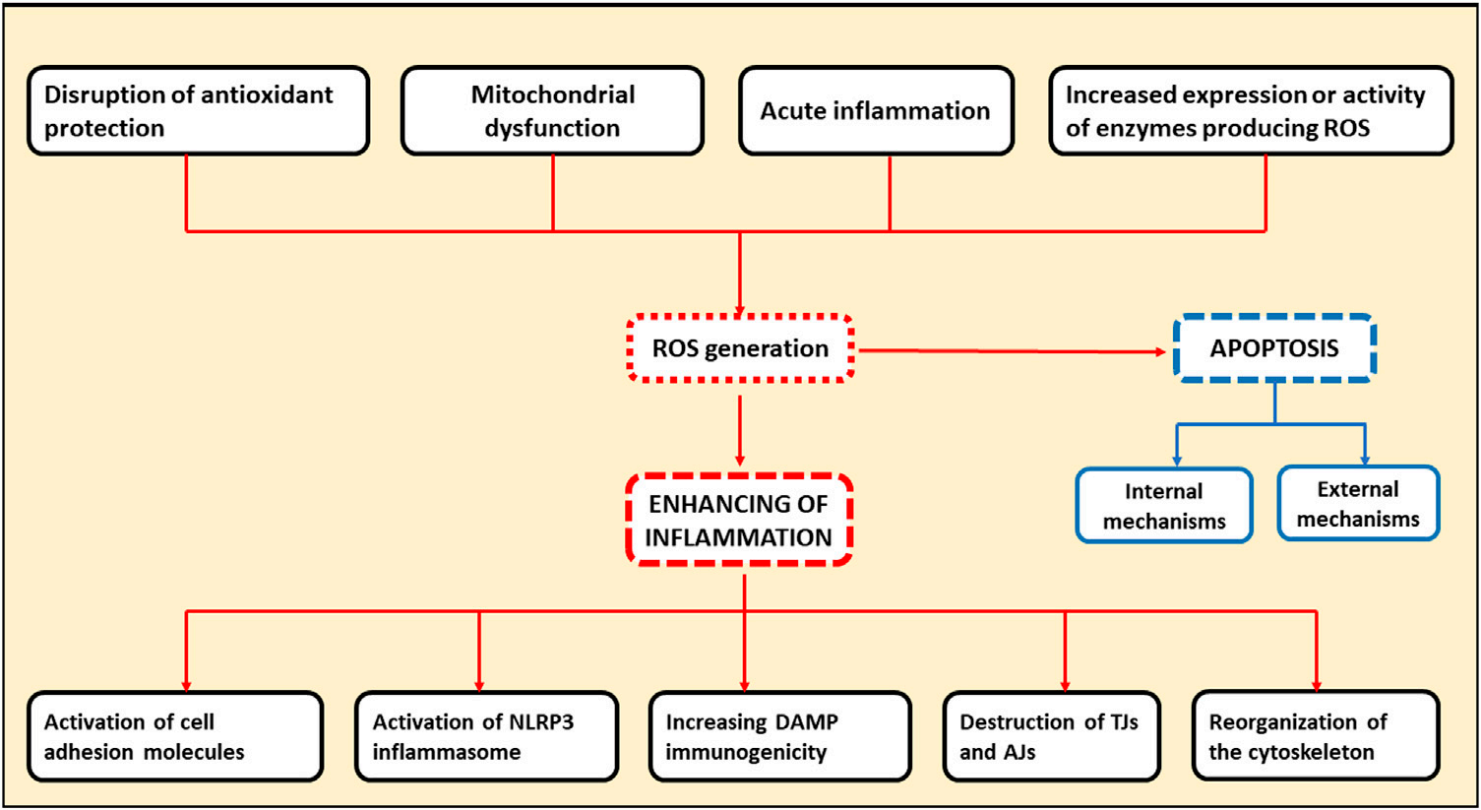
Schematic representation indicating the involvement of ROS in cellular apoptosis and chronification of inflammation. TJs, tight junction proteins; AJs, adhesion proteins.

ROS are directly involved in exacerbation of inflammation through several pathways. First, ROS can activate the NLRP-3 inflammasome, the one of the key complexes in innate immunity functioning. Inflammasome activation leads to the release of pro-inflammatory cytokines, such as IL-1 and IL-18, by leukocytes, further enhancing the inflammatory response (Mittal et al., 2014). Second, the increased production of ROS by the endothelial cells contributes to the activation of cell adhesion molecules located on the cell surface promoting extravasation of leukocytes into the site of inflammation. At the same time, ROS are able to activate cell adhesion molecules both as a result of direct interaction and via transcription factors such as nuclear factor kappa-light-chain-enhancer of activated B cells (NF-kB) and activator protein 1 (AP-1) (Mittal et al., 2014). Oxidative stress, resulting from the overproduction of ROS by leukocytes in the inflammation site, plays a major role in the destruction of adhesion proteins and tight junction proteins. This leads to gaps between cells, which facilitates the migration of immune cells and further tissue damage (Mittal et al., 2014). Additionally, dissociation between the endothelial cells can occur when the components of oxidative stress interact with actin cytoskeleton. In this case, reorganization of cytoskeleton can occur through direct oxidation and disruption of the cytoskeleton proteins leading to structural changes, or through inhibition of actin polymerization by interference with the signaling pathways involved in this process (Mittal et al., 2014). Infection-free (sterile) inflammation occurs when immune cells recognize damage-related molecular patterns (DAMPs) as antigens (Roh and Sohn, 2018). DAMPs are endogenous danger molecules that are secreted from damaged cells and stimulate the innate immune system by interacting with pattern recognition receptors. The inflammatory response itself contributes to the production of new DAMPs resulting from the destruction of cellular components in the course of inflammation process. Consequently, a positive feedback loop may form, promoting inflammation to persist and, ultimately, chronic inflammation. ROS contribute to this process by modifying DAMPs by oxidation, which increases their immunogenicity (Roh and Sohn, 2018). Therefore, ROS are directly involved in the initiation of inflammation and its subsequent chronification, which can occur when acute inflammation fails to resolve. General scheme of the mechanisms of ROS involvement in cellular apoptosis and chronification of inflammation is presented in Figure 1.

## 3 Cellular antioxidant enzymatic system

**FIGURE 2:**
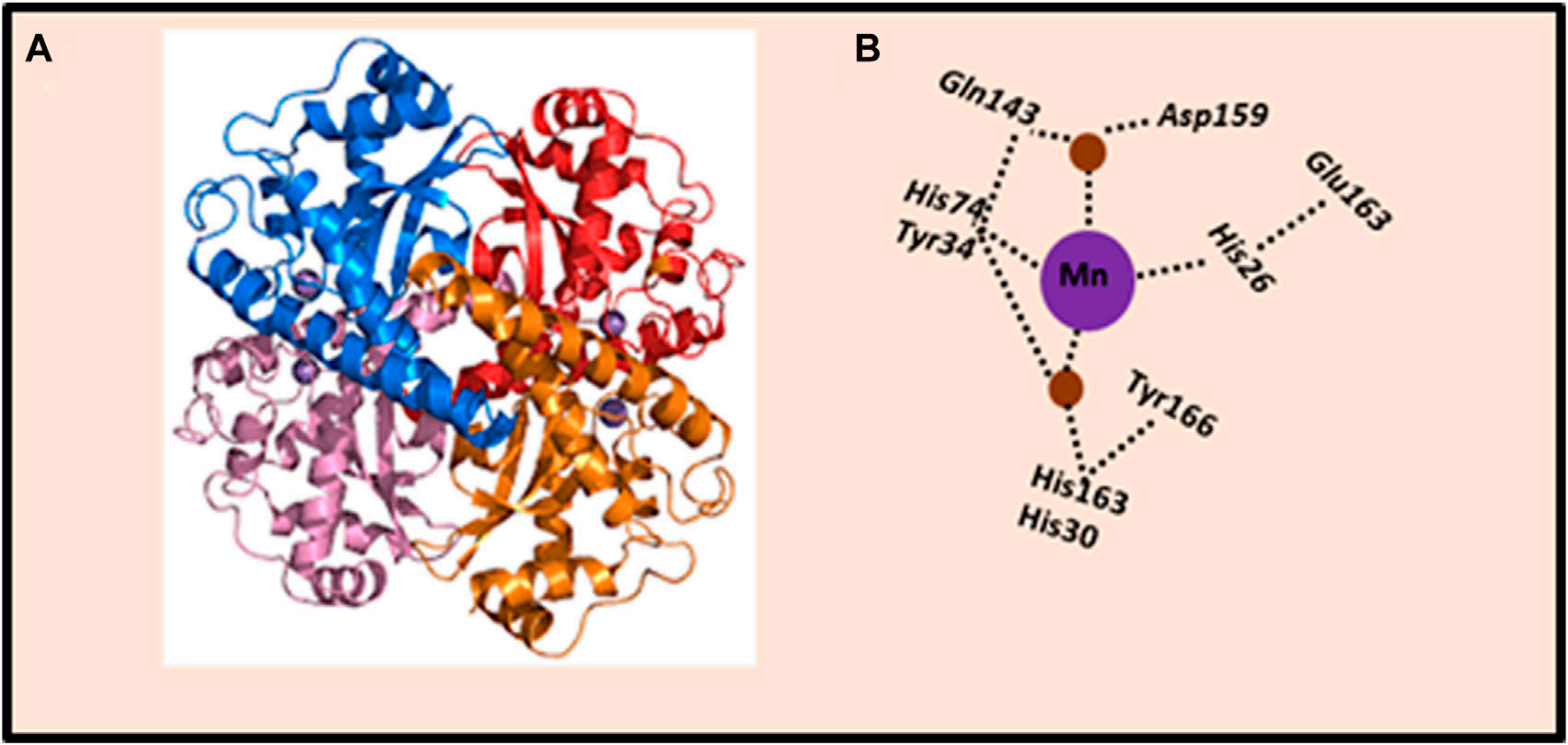
Crystal structure of human MnSOD. **(A)** Human MnSOD structure obtained from Protein Data Bank, code: 1LUV. Each monomer is shown in a different color; four subunits form MnSOD tetramer; each subunit contains a manganese ion at the catalytic center (active site) depicted as purple spheres. **(B)** Schematic representation of the active site of MnSOD and hydrogen-bonding. The dashed lines indicate the hydrogen bond network assumed to be the proton relay to the manganese ion utilized for catalysis. The side chains of active site residues His26, His74, His163, and Asp159 bind the manganese ion (depicted as purple sphere), joined with a water molecule (depicted as brown spheres). The water molecule forms a hydrogen bond with Gln143, and the network is extended with a hydrogen bond to Tyr34. Also, a water molecule mediates the hydrogen bond between Tyr34 and His30, which also forms a hydrogen bond with Tyr166 from an adjoining subunit. The figure was adapted from Bonetta (2018).

Cellular antioxidant enzymatic system that prevents the destructive effects of ROS, is represented by a number of enzymes that catalyze reactions aimed at converting ROS into harmless metabolites. The properties of the main antioxidant cellular enzymes, which include superoxide dismutase (SOD), catalase (CAT), glutathione peroxidase (GPx), glutaredoxins (Grx), and peroxyredoxins (Prx), have been long-established (Handy and Loscalzo, 2012). In particular, SOD represents a major defense against ROS. Three main isoforms of SOD are present in mammalian cells: (1) SOD1, the homodimer isoform, containing Cu and Zn atoms (Cu/ZnSOD), is present in the cytoplasm, nucleus, plasma, and inter-membrane space of mitochondria; (2) SOD2, the homotetramer isoform, containing the Mn atom in the active center (MnSOD), is located in the matrix and inner membrane of mitochondria; and (3) SOD3 or EC-SOD, the tetrameric glycoprotein isoform, is an enzyme associated with the cytoplasmic membrane and extracellular environment. SOD3 is secreted by the cell and binds extracellular O_2_•− (Stephenie et al., 2020). The metal ions that are commonly bound by SOD are iron (Fe), zinc (Zn), copper (Cu), and manganese (Mn). Of these, manganese-dependent SOD (MnSOD) plays a most important role because of its mitochondrial location, the main site of O_2_•− production (Figure 2).

**FIGURE 3:**
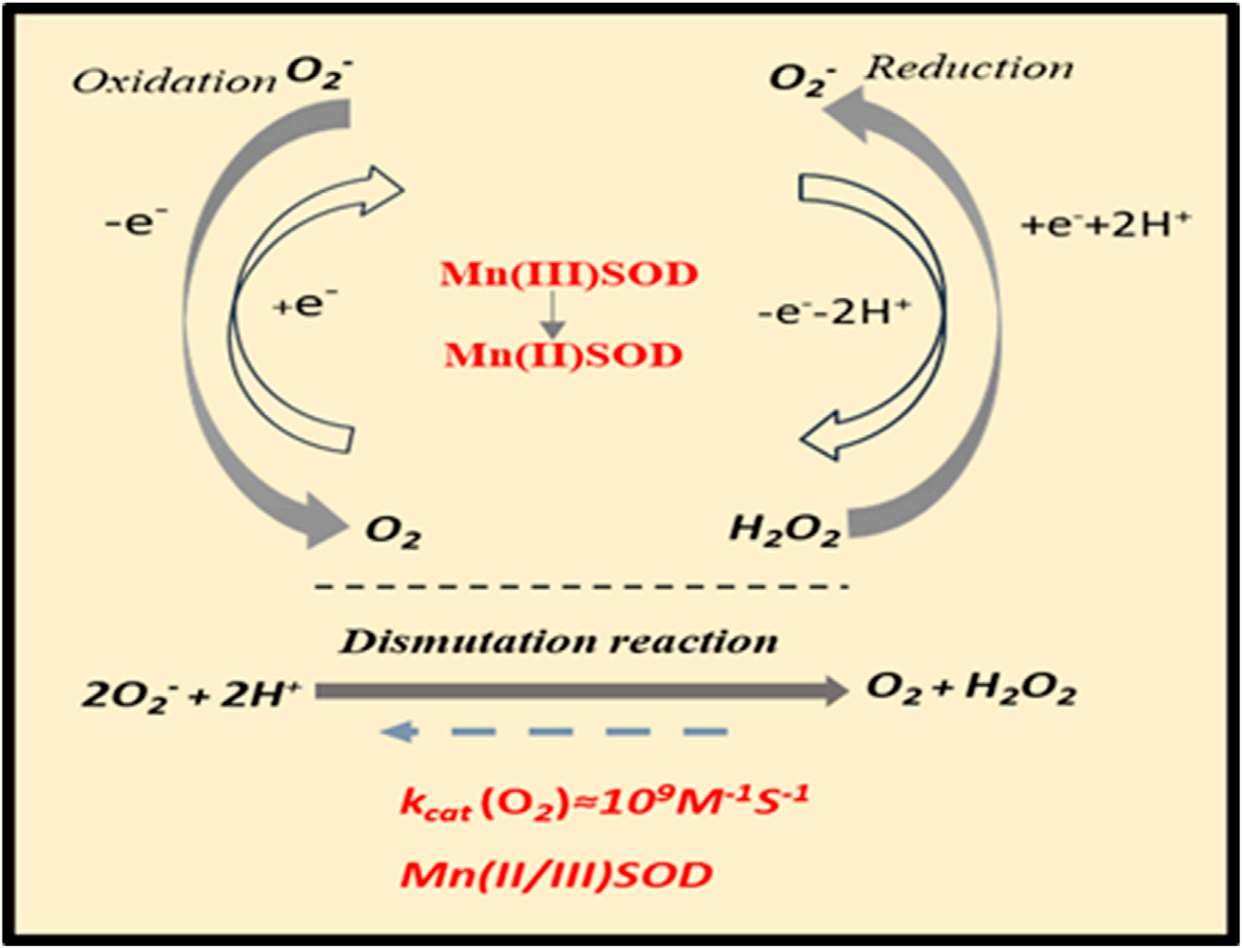
Dismutation reaction catalyzed by MnSOD.

Based on the SOD dismutation process, each isoform (Cu/ZnSOD, MnSOD, FeSOD) requires a redox transition metal for its appropriate function (Bresciani et al., 2015). Thus, MnSOD catalyzes the conversion of the O_2_•− free radical to H_2_O_2_ and molecular oxygen O_2_. The SOD-catalyzed dismutation reaction is highly efficient, which occurs at the almost diffusion-limited rate of ∼2 × 10^9^ M^−1^·S^−1^. Schematic representation of dismutation reaction catalyzed by manganese SOD (MnSOD) enzyme is shown in Figure 3. A complex role of MnSOD in forming cellular redox milieu and hence biological state of the cell was discussed based on thermodynamic and kinetic data (Buettner, 2011).

The dismutation of superoxide anion radical (O_2_•−) to hydrogen peroxide (H_2_O_2_) and molecular oxygen (O_2_) is catalyzed by MnSOD located in the mitochondrial matrix. The enzyme-catalyzed dismutation takes place approximately three orders of magnitude quicker than O_2_•− self-dismutation; it occurs at a potential, which is halfway between the potential for O_2_•− oxidation to molecular oxygen (O_2_) and its proton-dependent reduction to H_2_O_2_. In physiological conditions, H_2_O_2_ is removed in the next step by peroxide scavenging enzymes such as GPx and CAT. Abbreviations: CAT, catalase; GPx, glutathione peroxidase; MnSOD, manganese superoxide dismutase; k_cat_ (O_2_), rate constant for superoxide dismutation.

H_2_O_2_, formed during reactions involving SOD, is not only a central redox signaling molecule (mentioned above), but also a toxic metabolite. For its complete neutralization, a conversion of H_2_O_2_ to H_2_O is necessary, which is facilitated by CAT, localized mainly in peroxisomes, or GPx family of enzymes, members of which have distinct subcellular localizations (cytosol, nucleus, mitochondrial intermembrane space, extracellular matrix, and endoplasmic reticulum) (Buday and Conrad, 2021). Thus, the cell has a double degree of protection against the toxic effects of H_2_O_2_. GPx is also involved in the reduction reactions of lipid hydroperoxides (Pei and Pan, 2023). GPx contains selenium atoms in the active center, and CAT contains heme in its structure (Handy and Loscalzo, 2012). It is important to note that despite the fact that NADPH is not a direct cofactor of CAT, since it does not participate in the detoxification reaction of H_2_O_2_, it prevents inactivation of CAT by hydrogen peroxide and also helps to stabilize the molecule, thereby increasing the activity of the enzyme. Therefore, when oxidative stress occurs, NADPH plays an important role in maintaining the activity of CAT, as well as other antioxidant enzymes (Han et al., 2021). The reduction of NADP+ to NADPH occurs with the participation of various mitochondrial dehydrogenase enzymes, which are also important for the regulation of oxidative stress (Koju et al., 2022). The enzymes that decompose H_2_O_2_ to water also include peroxyredoxins (Prx). Currently, six types of this enzyme have been identified (Poole and Nelson, 2016). Despite the fact that Prx are considered to be less effective for H_2_O_2_ neutralization to water than CAT and GPx, they have a sufficiently low Michaelis constant value (Km < 20 mM) for H_2_O_2_, which enables rapid binding of H_2_O_2_ molecules (Wood et al., 2003). According to one hypothesis, Prx act not only as antioxidants but also as a regulator of the H_2_O_2_ concentration, which can serve as a signaling molecule inside the cell (Villar et al., 2023).

Since the endogenous antioxidant system governs both production and elimination of H_2_O_2_, a dual role, physiological and pathological, can be also identified for all the enzymes involved. Along these lines, the activity of SOD may have a double and opposite meaning. First, it may act as an antioxidant enzyme when its activity is controlled with either the CAT, GPx, or Prx enzymes, which prevent H_2_O_2_ accumulation by neutralizing it into H_2_O. Second, SOD may act as a pro-oxidant because H_2_O_2_ can build up, resulting in overproduction of ROS and cell toxicity (Wang et al., 2018).

Cellular antioxidant system is effectively regulated. Its main regulator is the transcription factor nuclear factor E2-related factor 2 (Nrf2), which is normally retained in the cytoplasm by the Kelch-like ECH-associated protein 1 (Keap1) protein, which also participates in proteosomal degradation of Nrf2 (Bellezza et al., 2018). Under oxidative stress conditions, with a decrease in pH, Nrf2 dissociates from *Keap1* and moves to the nucleus, where it dimerizes with transcription factors of the *MAF, ATF,* and *JUN* family and activates a complex of genes that have the antioxidant response element in their promoter regions. These genes encode antioxidant and cytoprotective enzymes (Mittal et al., 2014). Noteworthy, antioxidant enzymes are encoded by nuclear chromosomal genes, rather than mitochondrial genes, and transported to the mitochondria from the endoplasmic reticulum.

## 4 Antioxidants that potentially can be used for the development of drugs for the treatment of chronic inflammatory diseases

Exogenous antioxidants are useful when the body’s antioxidant system fails to combat high levels of ROS or when the system is defective. According to the Drug Bank online, more than 70 registered compounds with an antioxidant effect are currently used as pharmaceuticals or biologically active supplements. Details on these compounds can be found at (https://go.drugbank.com/categories/DBCAT000368). Moreover, more than 700 clinical studies related to antioxidant therapy are being conducted; details can be found at (https://classic.clinicaltrials.gov). Endogenous and exogenous antioxidants may exhibit synergistic action to maintain or restore redox homeostasis.

By their mechanisms of action, antioxidant compounds can be generally divided into the following groups: (i) substances that directly bind and restore ROS levels (the largest group); (ii) inhibitors of enzymes, such as NADPH oxidase, and myeloperoxidase, that are involved in ROS production; (iii) enhancers of antioxidant enzymes expression or artificial antioxidant enzymes; and (iv) molecules preventing the damage of macromolecules caused by the action of ROS (Aziz et al., 2019; Forman and Zhang, 2021). By their origin, antioxidant compounds are traditionally divided into natural (isolated from plants or other organic sources) and synthetic (obtained by chemical synthesis) (Aziz et al., 2019). The mechanisms of action and the effects of the most common natural and synthetic antioxidants are described in the next two subsections. It has to be noted that we did not try to cover all the options.

### 4.1 Natural antioxidants

Vitamins C and vitamin E are the most extensively studied antioxidants (Li et al., 2014). Vitamin C, also known as ascorbic acid, is a water-soluble substance found in various fruit and vegetables (Doseděl et al., 2021). The antioxidant effect of vitamin C is based on the reaction of reducing ROS, such as O_2_•− and singlet oxygen containing HOO− or OH− by dehydrogenation to form dehydroascorbate and reduced ROS products, such as water, as described in Li et al. (2014).

Due to the fact that vitamin C is water-soluble, it is able to protect cells from external ROS molecules present in the bloodstream (Li et al., 2014). Some studies had shown the protective role of vitamin C in patients with hyperlipidemia and essential hypertension (Shidfar et al., 2003; Schneider et al., 2005). Beneficial effect of vitamin C is primarily associated with the antioxidant protection of blood vessels, i.e., it reduced the content of low-density lipoproteins (LDL) that cause chronic inflammation-associated atherosclerotic vascular damage, the underlying cause of CVDs (Shidfar et al., 2003). More recent data obtained from both preclinical and clinical studies also showed that vitamin C plays an important role in several of processes involved in the pathogenesis of CVD (Morelli et al., 2020). Vitamin C can improve endothelial function, reduce NAD(P)H oxidase activity and superoxide production, and increase eNOS activity and production of nitric oxide (Morelli et al., 2020). In particular, endothelial dysfunction, characterized by reduced nitric oxide bioactivity, is an early sign of type 2 diabetes mellitus and is involved in the pathophysiology of atherosclerosis. A systematic review summarized clinical studies that reported the effects of vitamin C intake on CVD-related outcomes in people with metabolic syndrome and type 2 diabetes (Dludla et al., 2022) Some studies reporting positive effects are presented in Table 1.

**TABLE 1:** Clinical studies reporting the impact of oral vitamin C consumption on CVD-associated outcomes in people with metabolic syndrome and type 2 diabetes mellitus.

Country	Number of participants	Age of participants (years)	Vitamin C dose and period of intake	Main findings	References
United Kingdom	30	45–70	500 mg daily for 4 weeks	Lowered arterial BP; improved arterial stiffness	Mullan et al. (2002)
United Kingdom	20	Mean 53.2	1 g daily for 2 days	Improved endothelial dysfunction; attenuated lipemia induced post-prandial oxidative stress	Anderson et al. (2006)
Iran	84	Mean 53.2	1,000 mg daily for 6 weeks	A significant decrease in FBS, TG, LDL, HbA1c, and serum insulin	Afkhami-Ardekani and Shojaoddiny-Ardekani (2007)
Palestine	64	Mean 50.7	500 mg twice daily for 8 weeks	Reduced inflammatory markers hs-CRP and IL-6; reduced FBS and TG	Ellulu et al. (2015)
Australia	14	Mean 59.4	500 mg twice daily for 4 months	Significantly increased peripheral insulin sensitivity; ameliorated skeletal muscle oxidative stress during hyperinsulinaemia; improved insulin-mediated glucose disposal	Mason et al. (2016)
Australia	31	Mean 68.1	500 mg twice daily for 4 months	Improved post-prandial and 24-h glycaemia; lowered BP	Mason et al. (2019)

Adapted from Dludla et al. (2022). Abbreviations: FBS, fasting blood sugar; IL-6, interleukin; HbA1c, average blood glucose levels for the last 2–3 months; hs-CRP, high-sensitivity C-reactive protein; LDL, low-density lipoprotein; TG, triglycerides.

Apart ROS scavenging capacity, vitamin C has a more remarkable effect on inflammatory signaling in cells, where it can clearly reduce the expression of pro-inflammatory mediators, hence inhibiting the inflammatory response (Tan et al., 2005). The major known pro-inflammatory signaling pathway influenced by vitamin C is the nuclear factor κB/tumor necrosis factor alpha (NFκB/TNFα) pathway. Remarkably, three staple targets (phosphatidylinositol 4,5-bisphosphate 3-kinase catalytic subunit alpha isoform, signal transducer and activator of transcription-3 (STAT3), and prothrombin) of vitamin C with therapeutic potential for CVD have been identified (Zhu et al., 2021). This study showed that the protective effects of vitamin C on CVD were attributed to its anti-inflammatory property with the most relevant molecular pathways, such as the JAK/signal transduction and transcription activation, programmed cell death protein 1, the epidermal growth factor receptor, the Forkhead box O, and chemokines signaling pathways. In addition, the administration of vitamin C to patients with congestive heart failure (CHF) suppressed apoptosis in endothelial cells (Rössig et al., 2001; Aimo et al., 2020). Noteworthy, both systemic inflammation and increased oxidative stress are implicated in the pathogenesis of CHF.

Vitamin E, also known as tocopherol, is a fat-soluble compound contained in vegetable fats, the high contents of which were found in nuts and seeds (Rizvi et al., 2014). Unlike vitamin C, which primarily provides extracellular protection, vitamin E, contained in lipid membranes, which are the most common targets of ROS, inhibits lipid peroxidation (Rizvi et al., 2014). In addition, vitamin E prevents the oxidation of nucleic acids, directly removes O_2_•− and increases the activity of SOD, thus acting as an enhancer of the cellular antioxidant defense (Surai and Fisinin, 2015). Vitamin E was found to act as a scavenger of the peroxyl radical with the rate of reaction and scavenging much higher than the rate of peroxidation of lipid molecules by this radical (Khadangi and Azzi, 2019; Niki, 2021). The effects of vitamin E had been studied for potential treatment of CVDs, oncological diseases, as well as neurodegenerative diseases such as Alzheimer’s disease and Parkinson’s disease, reviewed in Li et al. (2014), Rizwana et al. (2022). In particular, the role of dietary vitamin E (α-tocopherol) as an antioxidant and bioactive molecule in supporting vascular health has been explored (Garg and Lee, 2022). Nevertheless, the protective function of vitamin E against CVD, for example, is not solely due to its ability to scavenge free radicals. A foremost part of its anti-inflammatory role occurs via the inhibition of NFκB (Rashidi et al., 2017) and reduction of the activity of protein kinase C involved in the endothelial cell adhesion molecule signaling (Cook-Mills, 2013). In this way, vitamin E can suppress atherogenesis and atherosclerosis development, the most common disease associated with oxidative stress and chronic inflammation. Moreover, the suppressive effect of vitamin E to tumors has been long-established. Early study using high performance liquid chromatographic analysis reported that the content of vitamin E in women with breast cancer is significantly lower than that in the cancer-free group, suggesting that vitamin E can reduce the risk of breast cancer (Sundaram et al., 1981). More recent study also found that alpha-tocopheryl succinate, the most efficient form of vitamin E, has tremendous anti-cancer potential (Wu et al., 2020). This study indicated that nanoparticle-delivered vitamin E in combination with interferon gamma (IFN-γ) represents a valuable strategy for cancer treatment. Combined application of nanoparticle-delivered vitamin E with IFN-γ significantly increased the anticancer effect in terms of apoptosis initiation and migration inhibition, as well as upregulated the anticancer immunity through the expression of downregulating program death ligand 1 (PD-L1). Besides, this combination therapy modulated the cytotoxic lymphocyte infiltration into the tumor microenvironment for its elimination. A review summarized a significant number of studies demonstrating the effects of vitamin E related to the suppression or overexpression of main tumorigenic pathways, mostly associated with breast cancer progression, proliferation, metastasis, tumor energy metabolism, and its effects in combination with chemotherapy drugs (de Oliveira et al., 2021). Nevertheless, conflicting results have been observed in randomized clinical trials evaluating vitamin E isoforms in human cancer treatment and pathophysiological processes associated with anticancer drugs (Donnelly et al., 2022). These results do not allow to make a meaningful conclusion on the beneficial role of vitamin E in cancer therapy.

A recent review summarized the scientific evidence published between 2000 and 2023 on the effects of vitamin E supplementation on neuroprotection and neurodegeneration markers in animal models of neurodegenerative diseases (da Cunha Germano et al., 2023). The studies considered in this review provided evidence that vitamin E supplementation can reduce oxidative stress (lipid peroxidation) and inflammatory process in the brain in murine models of neurodegenerative diseases. Increased brain oxidative stress, which chiefly manifests as lipid peroxidation, is a crucial feature of the Alzheimer’s disease. The pathogenesis of Alzheimer’s disease is also associated with soluble beta-amyloid (Aβ) oligomers (Bright et al., 2015) and neuroinflammation (Onyango et al., 2021). In Parkinson`s disease, oxidative stress and systemic inflammation also stimulate the development of neuronal dysfunction caused by specific elements, Aβ, α-synuclein, tau or prion (Forloni et al., 2021; Chakrabarti and Bisaglia, 2023). Table 2 presents some studies on the positive effects of vitamin E supplementation in animal models of Alzheimer’s and Parkinson’s diseases.

**TABLE 2:** Positive effects of vitamin E supplementation in animal models of Alzheimer’s and Parkinson’s diseases.

Animal model	Dose of vitamin E/administration route	Duration	Main findings	References
Tg2576 transgenic mouse model of Alzheimer’s disease	2 mg/g (orally)	8 months	Reduced Aβ levels and deposition	Sung et al. (2004)
Tg2576 transgenic mouse of Alzheimer’s disease	210 mg/kg (gavage administration)	15 days	Reduced oxidative stress resulting from senile plaques	Garcia-Alloza et al. (2006)
APPswe/PS1dE9 transgenic mouse model of Alzheimer’s disease	100 mg/kg (orally)	4 weeks	Decreased oxidative stress and the levels of Aβ oligomer in the brains of mice; prevented the production of iNOS and inflammatory mediators, such as interleukin-6 and interleukin-1β; inhibited microglial activation by inhibiting NF-κB signaling pathway	Wang et al. (2016)
Wistar rat model of Alzheimer’s disease	25, 50, and 100 mg/kg/day (orally)	14 days	Reduced congophilic amyloid plaque and neurofibrillary tangle density in the hippocampus of rats	Jahanshahi et al. (2020)
Wistar rat model of Alzheimer’s disease	200 mg/kg (orally)	10 days	Suppressed the expression of apoptosis markers and improved hippocampal long-term potentials impairment and the memory deficit induced by Aβ	Shahidi et al. (2021)
Healthy male rats of the Sprague Dawley strain	100 I.U/kg/day (intramuscular)	5 weeks	Reduced lipid peroxidation and improved levels of SOD and GSH and thus can be neuroprotective in Parkinson`s disease	Sharma and Nehru (2013)
C57BL/6 mouse model of Parkinson’s disease	50 mg/kg/day (orally)	15 days	Attenuated midbrain activity of COX-2 and oxidative stress markers (lipoperoxides and nitrite/nitrate)	Ortiz et al. (2013)
Wistar rat model of Parkinson’s disease	5 and 10 mg/kg (intraperitoneally)	40 days	Attenuated behavioral changes; improved the expression of neurotransmitters; reduced the levels of inflammatory markers; decreased MAO-A and MAO-B; improved complex-I, complex-IV, cAMP levels	Singh and Chauhan (2022)
Swiss albino mouse model of Parkinson’s disease	5, 10, 20, and 40 mg/kg (orally)	23 days	Increased the levels of antioxidant enzymes and neurotransmitters; decrease the levels of inflammatory cytokines and α-synuclein mRNA expression	Iqbal et al. (2022)

Abbreviations: Aβ, amyloid-β; cAMP, cyclic adenosine monophosphate; COX-2, cyclooxygenase 2; GSH, glutathione; iNOS, inducible nitric oxide synthase; MAO, monoamine oxidase; mRNA, messenger RNA; NF-κB, nuclear factor κB; SOD, superoxide dismutase.

The protective action of vitamin E on Alzheimer’s disease and Parkinson’s disease has been also studied in humans (Schirinzi et al., 2019; de Leeuw et al., 2020). The studies showed that vitamin E may be a potential, beneficial agent for patients with these diseases. Further, dietary intervention clinical trials are required to verify effects of vitamin E.

Vitamin C and E in combination have a synergistic antioxidant effect, which was manifested as an increase in the expression of antioxidant enzymes, such as CAT, SOD, and GPx (Soylu et al., 2006). The simultaneous use of vitamin C can support the effect of vitamin E in the treatment of cardiovascular (Jha et al., 1995; Ashor et al., 2014) and neurodegenerative diseases (Martin et al., 2002; Sindhu et al., 2022). As for the latter, the combined use of vitamins C and E targeting oxidative stress and mitochondrial dysfunction can be a useful strategy for the treatment of neurodegenerative diseases (Dumont et al., 2010; Wu et al., 2019). In particular, Nrf2/antioxidant response element (ARE) pathway is a promising antioxidant target to counteract mitochondrial dysfunction (Hegazy et al., 2019). Mitochondrial dysfunction is a prominent feature in the pathogenesis of neurodegenerative diseases. A meta-analysis revealed that combined supplementation with vitamins A and E resulted in a small but significant reduction in arterial stiffness (Ashor et al., 2014). An increased arterial stiffness correlates with CVD events (Sharif et al., 2019). The beneficial effects of these vitamins on vascular stiffness may be explained by the reduction of the detrimental effects of ROS on structural and functional components of the vascular walls (Plantinga et al., 2007). Antioxidant vitamins C and E neutralize ROS, diminish inflammation, and thus improve arterial elasticity and protect the integrity of the vessel wall (Kals et al., 2006). Furthermore, vitamin C can play a role in the prevention of endothelial dysfunction, an early sign of atherosclerosis and future cardiovascular events, since it is capable of preventing leucocytes adhesion to endothelial cells triggered by both oxidized LDL and cigarette smoke (Carr and Maggini, 2017). This effect of ascorbic acid is associated with the recovery of α-tocopherol (Doseděl et al., 2021). Oxidative modification of the protein portion of LDL can be caused by oxidative stress through ROS produced, for example, by leukocytes. In this case, the first line of antioxidant defense is α-tocopherol, which is the utmost antioxidant in LDL. However, the generated α-tocopherol radical can act as a prooxidant if not removed. Here, ascorbic acid appears to be of fundamental importance because it can reduce α-tocopherol to form the free radical ascorbate (ascorbyl), also known as monodehydroascorbate or hemidehydroascorbate (Carr and Maggini, 2017). The resulting ascorbate (ascorbyl) radical is relatively stable and can be detected in biological fluids in a concentration of 10 nM (Padayatty et al., 2003). The main reaction of this radical is the formation of dehydroascorbic acid. Also, it can be converted to ascorbic acid by NADH-dependent or NADPH-dependent enzymes, such as thioredoxin reductase and cytochrome *b5* reductase. There are molecular mechanisms by which vitamins C and E improve endothelial function (Ungvari et al., 2010), including the reversal of NF-κB-driven systemic chronic inflammation, which can accelerate aging through the ROS-mediated aggravation of telomere dysfunction and cell senescence (Jurk et al., 2014). Oxidative stress initiates the activity of redox-sensitive transcription factors, such as the activator protein (AP-1) and NF-κB, which enhances the expression of cytokines, adhesion molecules, and pro-inflammatory enzymes (El Assar De La Fuente et al., 2012). These factors promote the pro-inflammatory microenvironment and support the development of vascular dysfunction (Pashkow, 2011). Besides, both antioxidant vitamins C and E increase the bioavailability of nitric oxide, a vasodilator and anti-inflammatory substance (Correia and Haynes, 2007). However, a systematic review and meta-analysis of randomized controlled trials examining the effects of vitamins C and E supplements on endothelial function have yielded conflicting results (co-administration of these vitamins was ineffective) (Ashor et al., 2015).

The second group of known natural antioxidants includes carotenoids, including β-carotene, lycopene, and astaxanthin (Li et al., 2014). The direct interaction of carotenoids with ROS can be divided into three types of reactions: (i) electron transfer from the carotenoid molecule to ROS, (ii) dehydrogenation of the carotenoid, and (iii) ROS binding to the carotenoid molecule through the addition reaction (Li et al., 2014). Astaxanthin is also able to increase the activity of antioxidant enzymes SOD and GPx (Wu et al., 2014). The positive effects of β-carotene were reported in murine models of diabetic retinopathy, a low-grade chronic inflammatory disease (Kern, 2007; Kowluru et al., 2014). Lycopene and astaxanthin were investigated in pre-clinical settings for the treatment of neurodegenerative diseases (Liu et al., 2013; Ye et al., 2013; Min and Min, 2014). Other animal studies on the effects and molecular mechanisms of carotenoids in chronic inflammatory diseases are presented in Table 3.

**TABLE 3:** Effects of carotenoids in murine models of chronic inflammatory diseases.

Study	Disease model	Carotenoid dose/administration route	Duration	Main findings
Hira et al. (2019)	Alzheimer’s disease	β-carotene (2.05 mg/kg, orally)	14 days	Inhibited acetylcholinesterase activity; attenuated cognitive deficit via anti-oxidative action
Chen et al. (2019)	Alzheimer’s disease	β-carotene (10 mg/kg-50 mg/kg by gavage administration)	7 days	Alleviated oxidative stress by modulating the Nrf2/Keap1-mediated antioxidant pathway
Monroy-Ruiz et al. (2011)	CVD	Astaxanthin (75 or 200 mg/kg/day, orally)	8 weeks	BP lowering effect that is associated with improved endothelium-dependent vasodilatation and reduction of O_2_•− production
Park et al. (2015)	Diabetes type 1	Astaxanthin (50 mg/kg body weight/day, orally)	18 days	Attenuated hyperglycemia and thus inhibited both advanced glycation end product and ROS formation (lipid peroxidation), as well as inflammatory responses
Feng et al. (2020)	Gestational diabetes	Astaxanthin (10, 25, or 40 mg/kg body weight/day, orally)	3 weeks	Improved glucose uptake and glucose consumption through inhibiting production of inflammatory cytokines secretion (IL-1α, IL-6, TNF-α) and ROS
Canene-Adams et al. (2013)	Cancer	Special diet rich in lycopene	52 weeks	Markedly decreased the number and size of cribiform prostatic intraepitheilial neoplasia/carcinoma *in situ*; decreased the incidence of invasive intestinal adenocarcinomas and skin carcinomas

Abbreviations: BP, blood pressure; Keap 1, IL, interleukin; the Kelch-like ECH-associated protein 1; Nrf2, nuclear factor E2-related factor 2; O_2_•−, superoxide anion; TNF-α, tumor necrosis factor alpha.

There have been a great deal of human research on the role of carotenoids in chronic diseases. An evidence-based review focused on human clinical trials summarized and discussed astaxanthin favorable effects in Alzheimer, Parkinson, CVDs and cancer (Donoso et al., 2021). There are numerous human studies on the anti-cancer activity of lycopene, summarized in Kapała et al. (2022). Remarkably, lycopene benefit was verified by most of the studies. This review also provided the evidence obtained from multiple *in vivo* studies on molecular mechanisms involved in lycopene anti-tumor activity, including inhibition of angiogenesis via reduction of in vascular endothelial growth factor (VEGF) activity, reduction of oxidative DNA damage, enhancing cytotoxicity and cell apoptosis. In addition, a recent meta-analysis of randomized controlled trials arrived at the conclusion that β-Carotene supplementation has no beneficial or harmful effect on cancer incidence; however, it might have potentially damaging effects on lung cancer, especially in smokers (Zhang et al., 2023).

Polyphenols are another group of natural antioxidants (Singla et al., 2019). The antioxidant properties of its well-established representative, curcumin, a natural polyphenol component derived from *Curcuma longa*, were recently reviewed (Rizwana et al., 2022). The most common group of plant polyphenols includes flavonoids, flavones, isoflavones, anthocyanins, and xanthonoids (Li et al., 2014; Aziz et al., 2019). The main mechanism of action of flavonoids, for example, is based on the reduction of ROS and lipid radicals by dehydration (Shen et al., 2022). Flavonoids are also able to activate the expression of antioxidant enzymes such as SOD and GPx (Aziz et al., 2019). Several examples of antioxidant and anti-inflammatory effects of other natural plant-derived polyphenols are presented in Table 4.

**TABLE 4:** *In vivo* antioxidant and anti-inflammatory effects of several natural polyphenols in pathological conditions.

Polyphenol/Representative	Mechanism of action	Disease model	References
Flavone (apigenin)	PPARγ activation and regulation of macrophage polarization; shift of macrophages from proinflammatory M1 to the anti-inflammatory M2 phenotype; increase in production of anti-tumor cytokines IFNγ, TNFα, and granzyme B in the blood	Obesity Lung cancer	Feng et al. (2016) Jiang et al. (2021)
Isoflavone (genistein)	NF-κB inhibition, prostaglandin inhibition, inhibition of pro-inflammatory cytokines, inhibition of iNOS inhibition, inhibition of ROS and free radical scavenging activity	Chronic inflammatory disorders	Goh et al. (2022)
Anthocyanin (delphinidin)	Decrease in acetylcholinesterase, amyloid precursor protein, amyloid beta, and ROS overproduction in hippocampus	Alzheimer’s disease	Heysieattalab and Sadeghi (2020)
Xanthonoid (mangiferin)	Activation of the Nrf2/HO-1 antioxidant pathway, the PPARγ anti-inflammatory pathway via downregulating NF-κB, increasing the antiapoptotic protein Bcl-2, and decreasing caspase-3	Gastric ulcer	Mahmoud-Awny et al. (2015)

Bcl-2, B-cell lymphoma 2 gene; IFNγ, interferon gamma; iNOS, inducible nitric oxide synthase; HO-1, hemoxygenase 1; NF-κB, nuclear factor κB; Nrf2, nuclear factor E2-related factor 2; PPARγ, peroxisome proliferator-activated receptor gamma; TNFα, tumor necrosis factor alpha.

The distinctive antioxidant mechanism of polyphenols action is the inhibition of enzymes responsible for the synthesis of ROS. Thus, it was shown that polyphenols could inhibit the expression of the NADPH oxidase subunits p22phox and p67phox, therefore acting as an inhibitors of ROS generation at the onset of the inflammatory reaction (Aziz et al., 2019). Polyphenols exhibited favorable an antioxidant effect in chronic inflammation-related diseases, such as alcoholic fatty liver disease, atherosclerosis, coronary heart disease, Alzheimer’s disease, and type 2 diabetes (Li et al., 2014).

Natural antioxidants have other biological functions unrelated to their antioxidant activity. For instance, vitamin C is primarily acts as a cofactor of an enzyme involved in the reaction of collagen formation (Institute of Medicine Committee on International Nutrition--Vitamin C in Food Aid Commodities, 1997). Vitamin E, in addition to suppressing oxidative stress, inhibits platelet aggregation and enhances both humoral and cellular immunity in infectious diseases (Rizvi et al., 2014).

Overall, natural oxidants hold robust potential for the prevention and therapy of diseases with an inflammatory, redox, or malignant component.

### 4.2 Synthetic antioxidants

New synthetic compounds with increased antioxidant properties have been developed in recent years (Salehi et al., 2018; Parcheta et al., 2021). Some of the synthetic antioxidants have been tested in different phases of clinical trials, the results of which have been discussed (Barteková et al., 2021). The results of these studies may justify a competitive advantage of new antioxidants over already approved drugs and dietary supplements. However, further safety check should be carried out carefully because of the possibility of some antioxidants at certain concentrations to cause the effect opposite to their main activity, i.e., the oxidative stress (Sotler et al., 2019).

**FIGURE 4:**
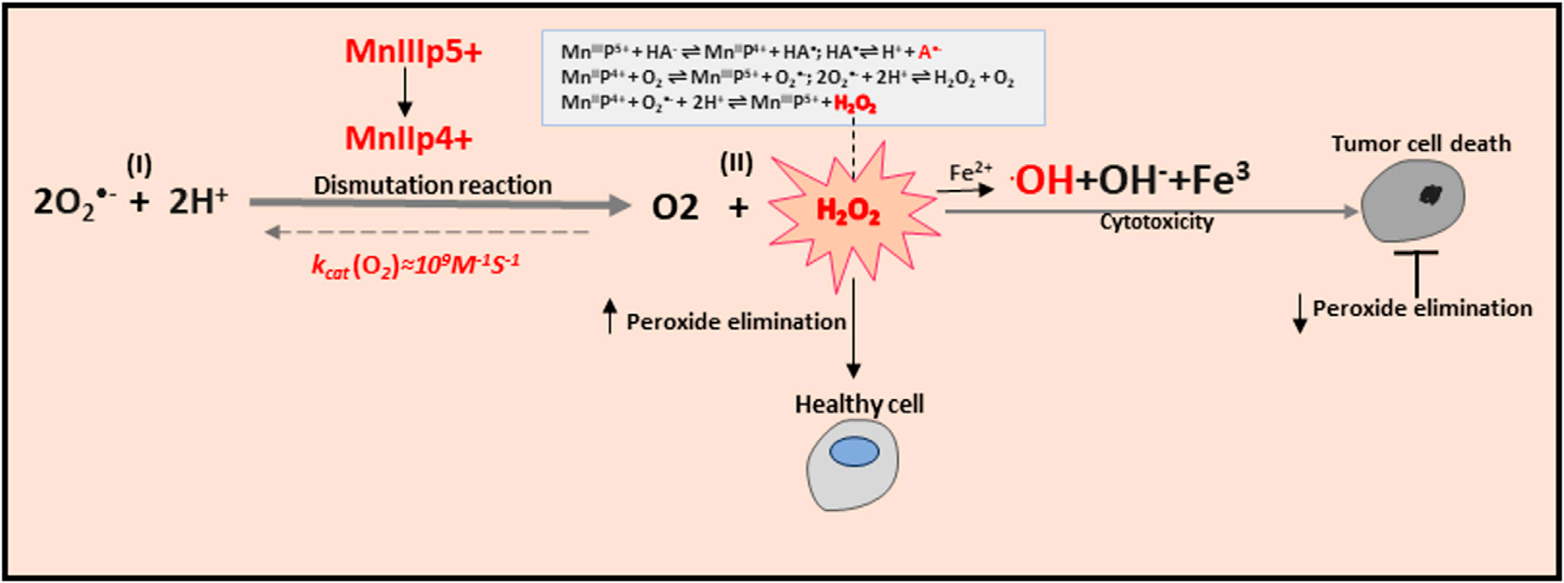
Anti-and pro-oxidative effects of MnPs.

Expanding understanding of the role of mitochondria in physiological and pathological conditions is paralleled by expanding research into compounds that can mimic SOD, the endogenous antioxidant defense of mitochondria necessary for the existence of almost all eukaryotes. It is worth mentioning that the use of SOD as a therapeutic agent has significant disadvantages, as follows: (i) the large size of the molecule, which prevents penetration into the cell, (ii) a short half-life in the bloodstream, (iii) the increased immunogenicity and high costs for protein production (Bonetta, 2018). Mimetics of SOD are a class of antioxidants, which resemble the activity of this enzyme by initiating the reaction of converting the superoxide anion into H_2_O_2_ (i.e., the dismutation reaction), the sequential reduction/oxidation of metal ions (Barteková et al., 2021). In the structure of almost every SOD mimetic, there is an active center containing a metal atom that directly participates in the reaction (Zhao et al., 2021). There are several classes of compounds used as SOD mimetics: cyclic manganese polyamines, metallic porphyrins, salen-manganese complexes, fullerenes, nitroxides, and metal nanoparticles (Li et al., 2020; Forman and Zhang, 2021). In particular, Mn-based SOD mimics such as the Mn(III) porphyrins (MnPs) have been vigorously studied (Batinic-Haberle et al., 2018). The most potent MnPs can oxidize and reduce O_2_•− with close to identical rate constants of SOD (>10^7^ M^−1^·s^−1^) (Batinic-Haberle et al., 2015). Remarkably, SOD-like MnPs exhibit both anti- and pro-oxidative activities *in vivo*, which may lead to either anti- or pro-oxidative (in cancer cells) therapeutic outcomes. MnPs counteract oxidative stress under physiological conditions, supporting normal tissue survival, and promote apoptotic processes in tumors and normal tissues under conditions of increased oxidative stress (Batinic-Haberle et al., 2018). MnPs can only be considered as antioxidant protection in combination with H_2_O_2_ removal systems. A growing body of evidence indicates that such systems fail in cancer cells. Reports suggest a decrease or inactivation of GPx and Prx enzymes with a concomitant increase in SOD levels during cancer progression (Celic et al., 2014; Tovmasyan et al., 2014). In turn, H_2_O_2_ is accumulated and utilized by a tumor cell for its proliferation; under such conditions, MnPs increase oxidative stress and can no longer be considered as an antioxidative defense (Batinic-Haberle et al., 2014). It was demonstrated that H_2_O_2_ is generated through cycling of MnP with ascorbate (Tovmasyan et al., 2016; Batinic-Haberle et al., 2021), which can be further metabolized to the hydroxyl radical (˙OH) via the Fenton reaction (Fe^2+^ + H_2_O_2_ → Fe^3+^ + ˙OH + OH^−^), the second part of the Haber-Weiss mechanism (Kremer, 2019) (Figure 4). In this way, MnPs can accelerate the rate of ascorbate radical oxidation, enhancing H_2_O_2_ flux and causing ascorbate-induced cytotoxicity. This redox cycling is the foundation of the therapeutic potential of the combination of MnPs/ascorbate for the treatment of cancers. Of note, MnPs can react *in vivo* with other redox active species, including glutathione, cysteines, H_2_O_2_, ONOO^−^, ^•^NO, tetrahydrobiopterin, hypochlorite (ClO^−^), and sulfite (SO_3_^2−^), as well as act as catalytic (redox-cycling) agents by catalyzing protein S-glutathionylation (Batinic-Haberle and Tome, 2019). Therefore, MnPs have the potential to universally modulate redox regulatory signaling networks in cells.

MnPs oxidize O_2_•− in step one (I) and reduce it in step two (II); both reactions undergo with rate constants (k_cat)_ identical to that of SOD enzymes. Dismutation of O_2_•− by MnPs involves MnIII/MnII redox couple. The dismutation reaction demonstrated here conveys the important information that SOD mimics MnP exhibit pro- and anti-oxidative actions during the dismutation of the O_2_•−. MnPs catalyze ascorbate oxidation, producing H_2_O_2_. In the presence of abundant peroxide removal systems, this activity will result in an antioxidant effect. However, if peroxide-removing enzymes are scarce, the H_2_O_2_ levels may rise and initiate cellular transcription, which would sequentially perpetuate oxidative stress. In these conditions, the ability of MnPs to catalyze ascorbate oxidation would promote oxidative stress. The metal site of MnP redox cycles between Mn + 3 and +2 oxidation steps simultaneously transferring an electron to ascorbate and forming ascorbyl radical, A^−^. The reoxidation of MnIIP to MnIIIP may take place with O_2_ or O_2_•−, and H_2_O_2_ would be ultimately generated. The resulting H_2_O_2_ may generate the hydroxide ion HO^−^ via the Fenton reaction in the presence of Fe^2+^. Production of H_2_O_2_ depends on the MnIII/MnII reduction potential and is needed to increase tumor cell death. MnP potency in catalyzing ascorbate oxidation is in a direct relationship with MnP/ascorbate-induced cytotoxicity. Notably, anti- or pro-oxidative *in vivo* effects of MnPs will depend upon the following conditions: (i) the cellular ROS levels, (ii) endogenous antioxidants, (iii) oxygen availability, superoxide/peroxide-removing enzymes ratio, and (iv) MnPs redox potential, their localization in a cell, and bioavailability.

**FIGURE 5:**
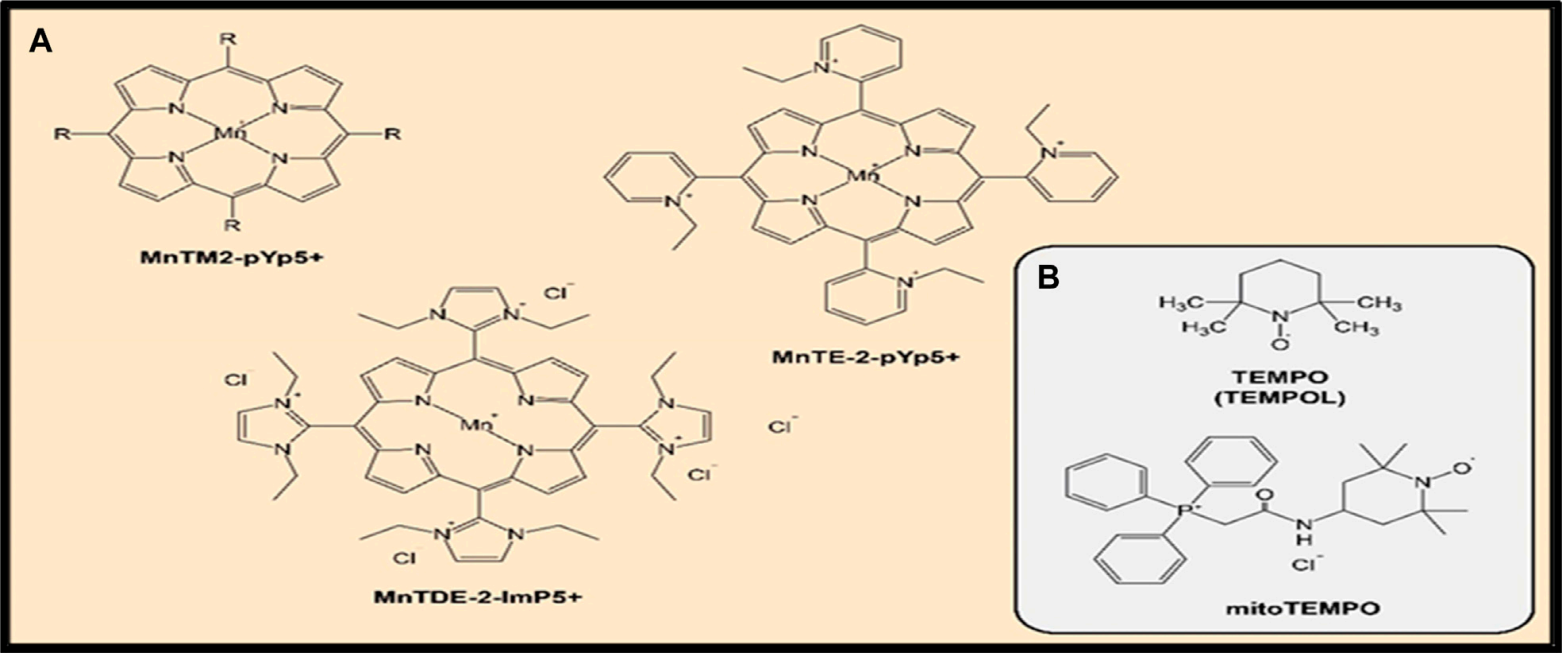
**(A,B)** Chemical structure of SOD mimics.

A review summarized the evidence indicating that SOD mimetics have a significant efficacy in several animal models that mimic oxidative stress injuries (Bonetta, 2018). Several compounds have been synthesized and investigated for their O_2_•− dismutation activity and some of them, such as Mn(III) meso-tetrakis (N- methylpyridinium-2-yl)porphyrin (MnTM2-pYp5^+^) and Mn(III) meso-tetrakis(N-ethylpyridinium-2-yl)porphyrin (MnTE-2-pYp5^+^), demonstrated a powerful SOD activity (Batinic-Haberle et al., 2015). Both protective and therapeutic effects of MnTE-2-pYp5^+^ and also Mn(III) meso-tetrakis (di-N-ethylimidazole) porphyrin (MnTDE-2-ImP5^+^) have been shown in animal models of inflammation-associated conditions such as breast tumor (Rabbani et al., 2009) and cardiovascular system damage (Ganesh et al., 2016). Moreover, MnTE-2-PyP5^+^ was found to amend oxidative stress-induced inflammation and enhance insulin sensitivity and glucose tolerance in a high-fat diet-induced mouse model of type 2 diabetes (Coudriet et al., 2017). These effects were attributed to ROS scavenging capacity of MnTE-2-PyP5^+^, resulting in a decrease in the pro-inflammatory cytokine secretion, by inhibiting of the action of NF-κB. The increased oxidative stress and inflammation play a chief role in the etiology of insulin resistance and type 2 diabetes (Henriksen et al., 2011). Figure 5A shows chemical structures of mentioned above MnP compounds.

One of the best-known representatives of SOD mimetics is the drug 4-Hydroxy-TEMPO (TEMPOL), which had shown effectiveness in a number of preclinical and clinical studies of the treatment of autoimmune, cardiovascular diseases, and oncological diseases (Bonetta, 2018; Barteková et al., 2021). TEMPOL belongs to the class of nitroxides and, unlike most SOD mimetics, the active component of the reaction is not a metal ion, but an oxoammonium cation (Bonetta, 2018). Additionally, TEMPOL can be used in the treatment of pain conditions; the ability of TEMPOL to suppress thermal and mechanical hyperalgesia has been demonstrated (Kim et al., 2016).

The elimination of ROS in the mitochondria themselves and thereby preventing the oxidative damage of cells at the earliest stages is also a strategy of the antioxidant therapy. A study demonstrated that the drug OP2113 can reduce the production of O_2_•− in the mitochondrial complex I of electron transport chain without having a negative effect on the ATP production (Detaille et al., 2019). Moreover, one approach that selectively block mitochondrial oxidative damage and prevent some types of cell death in order to develop mitochondria-targeted antioxidants was investigated (Murphy, 2008). In this respect, it was demonstrated that targeting bioactive molecules to mitochondria by conjugating them to lipophilic cations is a useful method (Murphy, 2008). The drug mitoTEMPO, which is a nitroxide of piperidine Tempo (a derivative of TEMPOL, mentioned above) with an attached lipophilic triphenylphosphonium cation is one characteristic example of mitochondria-targeting synthetic antioxidant compounds (Trnka et al., 2008). MitoTEMPO showed a greater effectiveness in the treatment of hepatotoxicity in a mouse model compared to TEMPO (Du et al., 2017). Also, it was shown that mitoTEMPO reduced the state of cardiomyopathy in mice with type 1 and type 2 diabetes (Ni et al., 2016). Diabetic cardiomyopathy is linked to chronic inflammation (Jia et al., 2018). Figure 5B shows chemical structures of the reported SOD mimic drugs.

In addition, the control of the mitochondrial oxidative stress, in particular, modulation of mitochondrial glutathione levels can have an impact on the disease progression, and therapies aimed at increasing mitochondrial glutathione levels have great potential for the treatment of numerous inflammatory diseases (Marí et al., 2009). A more recent study describes the use of the S-D-lactoylglutathione to supply mitochondria with mitochondrial glutathione, including the parent molecules that generate mitochondrial glutathione while located within the mitochondrial matrix (Armeni et al., 2014).

Another candidate for potential antioxidant therapeutic agents is nanoparticles with improved pharmacokinetics and high biological activity. Based on their different mechanisms of action, ROS-scavenging nanoparticles can be divided into the following categories: 1) nanozymes, 2) free-radical trapper nanoparticles, and redox ROS-scavenging nanoparticles. Several representatives of these nanoparticles are presented in Table 5.

**TABLE 5:** Several representatives of ROS-scavenging nanoparticles.

Enzymatic nanoparticles	Free-radical trapper nanoparticles	Redox ROS-scavenging nanoparticles
Cerium	Fullerene	Curcumin-based
Platinum-based	Tempo	Bismuth-based
Lead-based		
Copper-based		

Nanoparticles that had shown their effectiveness in the treatment of ROS-associated inflammatory diseases in preclinical studies include fullerenes, metal oxide nanoparticles (cerium, manganese, vanadium), and metal nanoparticles (platinum, vanadium, zirconium, molybdenum, manganese) (Li et al., 2020). Moreover, recent advances in ROS scavenging-based nanotherapeutics research and the mechanisms underlying the applied nanomaterial s have been thoroughly reviewed (Huang et al., 2021). In addition, adeno-associated virus mediated SOD gene therapy can be used to alleviate oxidative stress and improve mitochondrial dysfunction, the key features of inflammatory conditions (Jiang et al., 2016). This can be especially effective in the treatment of chronic diseases, characterized by low-grade oxidative stress and inflammation, where long-term treatment is required.

**FIGURE 6:**
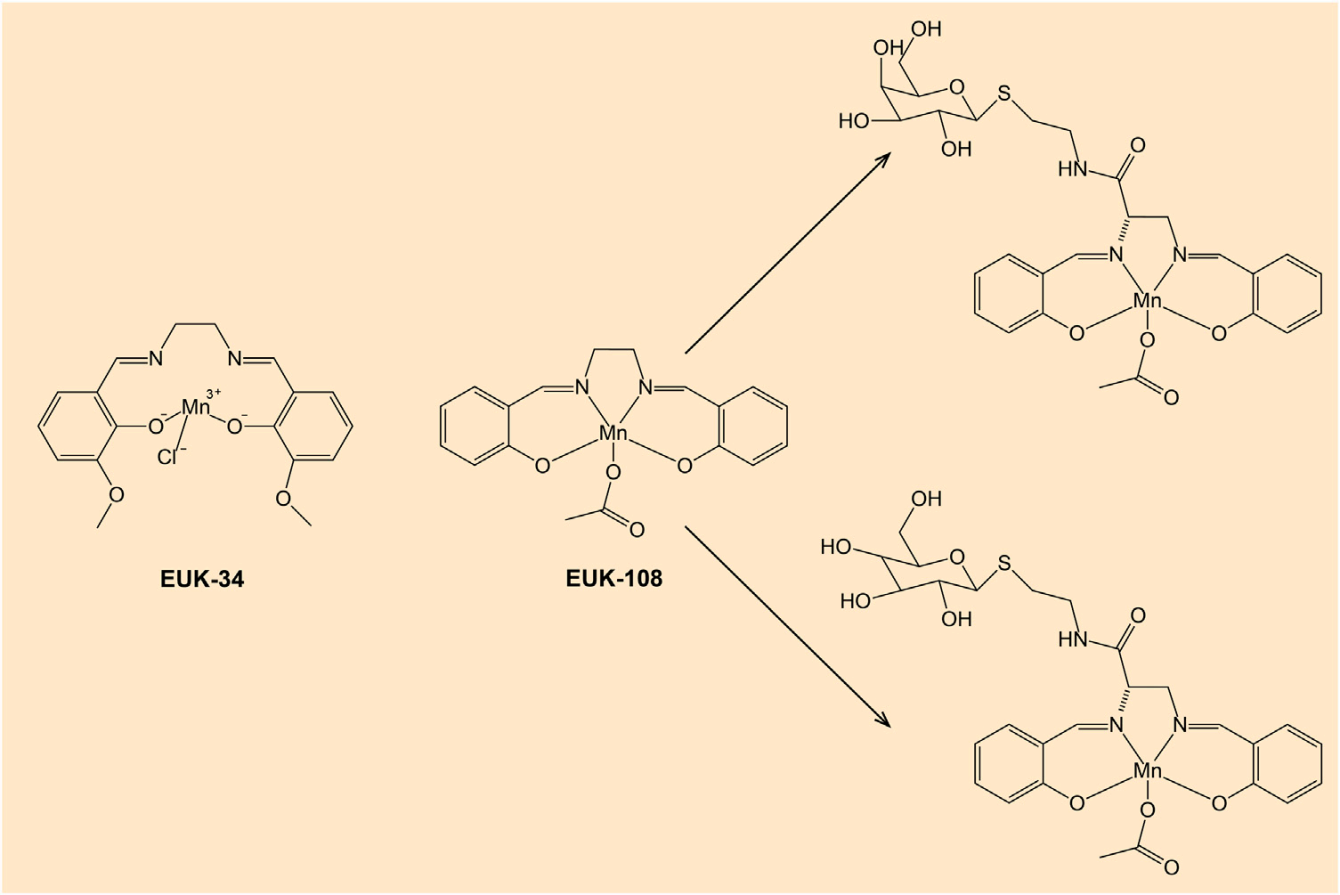
Chemical structures of EUK-34 and EUK-8 with glucose- and galactose-salen conjugates. Note: EUK-134 is an analog of EUK-8 with enhanced activity of CAT and the same SOD activity (Doctrow et al., 2002).

According to review data, a promising class of compounds are salen-manganese complexes, which mimic both SOD and CAT activities with broad pharmacological efficacy (Doctrow et al., 1997). Preclinical studies showed that salen-manganese complexes are effective in oxidative-stress-related conditions (Jung et al., 2001; McDonald et al., 2003; Rouco et al., 2020). In particular, Mn-3-methoxy N, N′-bis (salicylidene) ethylenediamine chloride (EUK-34) is a cell permeable mimetic of SOD and CAT enzymes capable to counteract redox stress (Himori et al., 2017). At present, EUK-134 is a component of many anti-ageing skincare products due to its antioxidant properties. Moreover, mitochondrial targeted antioxidant such as Mn(salen) chloride (EUK-8), another SOD and CAT mimetic, exhibited a strong potential to become a multi-target inhibitory molecule capable of mitigating inflammatory processes and thus to be a greater choice for the development of a powerful anti-inflammatory drug. In an *in vivo* experiment, EUK-8 significantly reduced the phosphorylation level of several inflammatory proteins such as p38 member of MAPK pathway, protein kinase B (Akt), and transcription factor p65, and inhibited pro-inflammatory cytokine interleukin-6 and chemokine CXCL1 (Jayachandra et al., 2023). A recent study reported a synthesis of new glycosalen-Mn(III) complexes of EUK-108 with glucose and galactose (Lanza and Vecchio, 2023). These complexes showed both SOD- and CAT-like activities and could be advantageous for future clinical applications. Notably, both antioxidant and prooxidant properties of EUK-8 have been determined (van Empel et al., 2006; Amaral and Espósito, 2008). Figure 6 shows chemical structures of EUK-34 and EUK-8 with glycoconjugates.

**FIGURE 7:**
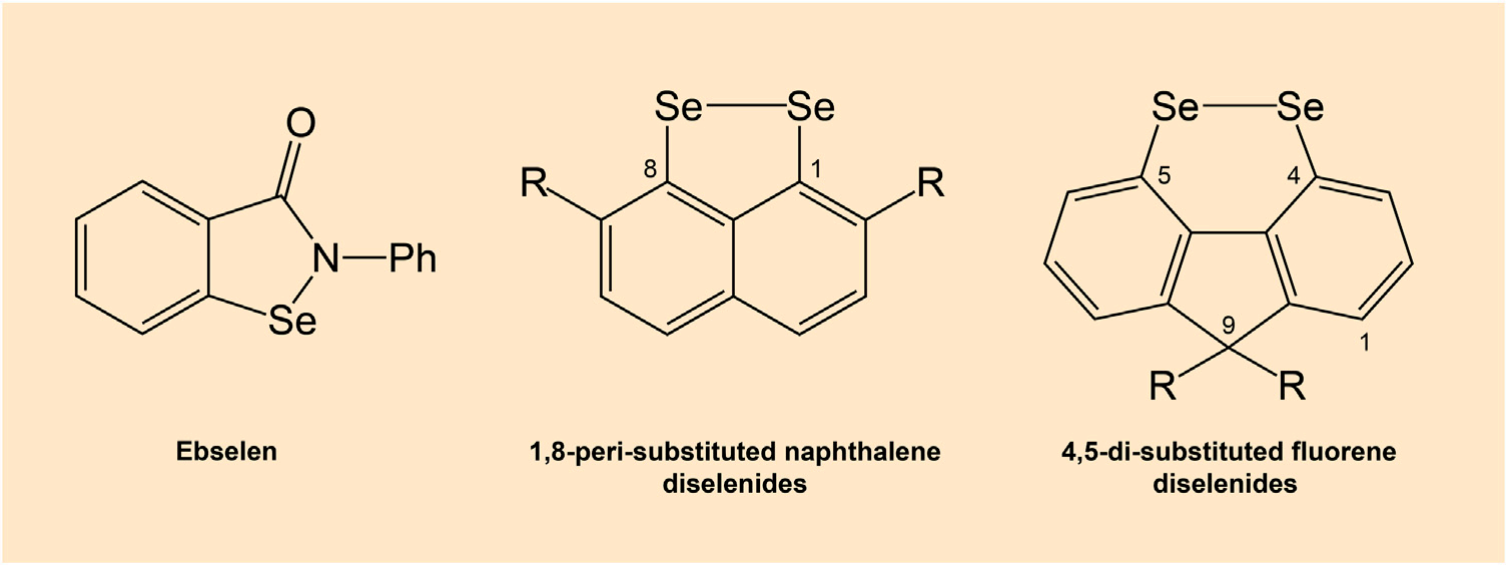
Chemical structures of ebselen and diselenides. Note: Diselenides are conformationally-restricted around the diselenide bond compounds.

There are compounds that mimic the activity of selenocysteine-containing GPx enzyme. In particular, diselenides are capable GPx mimics (Arai et al., 2021). Several organoselenium compounds such as ebselen and diselenides, which can mimic GPx activity hold a potential in drug development (Figure 7) (Nogueira et al., 2021). Various GPx mimics encompassing diverse selenium functionality have been reviewed, with the aminoselenide ebselen reaching phase 3 clinical trials for a number of diseases associated with oxidative stress (Barbosa et al., 2017; Sands et al., 2018).

In addition, the antioxidant properties of other compounds such as melatonin and lactic acid that can be utilized for the treatment of neurodegenerative diseases (Alzheimer’s disease and Parkinson’s disease) have been described in the review of Rizwana et al. (2022). Their review summarized the evidence indicating that melatonin can reduce the harmful effects of ROS by reducing the activity of NOS and stimulating antioxidant enzymes, such as SOD, GPx, and glutathione reductase, and lactic acid has prominent free radical scavenging effect.

The inhibition of NADPH oxidase, a ROS-producing enzyme, is an alternative approach to the antioxidant therapy. The inhibition of NADPH oxidase can be achieved through inhibition of catalytic activity and disruption of the assembly of NADPH oxidase complex consisting of several protein subunits (Forman and Zhang, 2021). To date, a number of synthetic compounds with high inhibitory activity against NADPH oxidase and therapeutic efficacy in the treatment of inflammatory diseases in animal models have been described (Altenhöfer et al., 2015). In particular, an individual NOX inhibitor VAS2870 has had a positive effect on experimental disease models associated with chronic inflammation such as stroke (Kleinschnitz et al., 2010) and Alzheimer’s disease (Abubaker et al., 2019).

The Nrf2 pathway is the main signaling pathway regulating the synthesis of antioxidant enzymes, which plays pivotal roles in inflammation (Ahmed et al., 2017). Therefore, the activation of the Nrf2 pathway is the most comprehensive and reliable measure of antioxidant and anti-inflammatory protection in comparison with other therapeutic strategies described above. According the mechanism of action, Nrf2 activators can be divided into the following categories: (i) activators of the transcription/translation of Nrf2, (ii) inhibitors of the Keap1 protein that binds and degrades Nrf2, (iii) inhibitors of other Nrf2 suppressors, such as glycogen synthase kinase-3 beta (in the cytoplasm) and (iv) Bach1 (in the nucleus), and (Forman and Zhang, 2021). To date, the most successful Nrf2 activator is fumaric acid ester dimethyl fumarate (DMF), which was approved in 2013 by the United States Food and Drug Administration (FDA) and the European Medicines Agency as a first-line therapy for adult patients with relapsing-remitting multiple sclerosis (immune-mediated inflammatory disorder) (Xu et al., 2015). A randomized, double-blind, placebo-controlled phase 3 study involving patients with relapsing–remitting multiple sclerosis from 198 sites in 28 countries was previously conducted (Gold et al., 2012). These data are also consistent with results from preclinical studies, in which DMF has been demonstrated to have favorable effects in animal models via direct neuroprotective and immunomodulatory mechanisms (Ghoreschi et al., 2011; Linker et al., 2011). The summary of fumaric acid esters, which were either approved for human use or tested in human or animal trials, was presented in the review of Kourakis et al. (2020). The main risk of therapeutic use of Nrf2 activators is associated with their possible impact on other signaling pathways and disruption of related biological functions. For example, sulforaphane exposure caused cell cycle arrest as a result of inhibition of the PI3K/Akt and MAPK/extracellular signal-regulated kinase (Erk) signaling pathways (Roy et al., 2010). Nevertheless, there have been significant advances in drug development to overcome systemic side effects of Nrf2 activators (Satoh and Lipton, 2017).

## 5 Discussion

Antioxidants are one of the promising groups of substances for the treatment of chronic inflammatory diseases. Both the search for new natural compounds and the development of synthetic drugs are currently relevant. However, antioxidant therapy faces challenges, including issues with bioavailability, appropriate dosing, and the complex interplay of multiple antioxidants in the body. The development of antioxidant drugs that directly bind ROS may be a dead end since their effectiveness is much lower in comparison with those targeting the cellular antioxidant system. New drugs should have high specificity to avoid systemic side effects, have good pharmacodynamic characteristics for long-term preservation of the pharmacological activity of the molecule and effective delivery to the target, as well as prohibit the development of a pro-oxidant activity, which may be characteristic of some antioxidants. One of the problems is the selection of the correct therapeutic concentration, on the one hand, sufficient to achieve effective action *in vivo*, and on the other hand—not to be in excess to avoid serious side effects. It should also be taken into account that for the complex treatment of chronic inflammatory diseases, different groups of antioxidant drugs may be required in order to block the production of ROS at various stages of inflammation: 1) drugs acting in the mitochondria; 2) in the cytoplasm; 3) in the bloodstream. At the same time, it should be understood that taking antioxidants that combat oxidative stress at various stages of development may not be enough to cure the disease due to the involvement of other signaling pathways not associated with ROS in inflammation. Besides, responses to antioxidant therapy may vary among individuals, and there is a need for personalized approaches based on genetic, environmental, and lifestyle factors.

Owing to the preclinical effects of antioxidants, a large number of clinical trials investigating whether antioxidants can be beneficial for the prevention and/or treatment of chronic inflammatory diseases have been conducted around the world in the past 3 decades. However, the human trials produced inconclusive results; some trials have demonstrated positive effects (Imamura et al., 2017), while others revealed either detrimental effects (Lonn et al., 2005) or no effects at all (van der Made et al., 2015). The definite reason for the failure of antioxidants to yield favorable effects in human inflammatory pathologies associated with oxidative stress remains unclear. Several researches suggested a few explanations to clarify the inconsistency between the findings of clinical trials and experimental studies, discussed in Biswas (2016). The first explanation is that oxidative stress is not a culprit in the pathogenesis of various chronic diseases and therefore the antioxidants are ineffective. This explanation is confusing as there is a great deal of literature elucidating the substantial contribution of oxidative stress to conditions related to chronic inflammation (Sun et al., 2020). We believe that it is important to recognize the limitations that have led to failures in clinical trials and how antioxidant defense can be effective if there is a clear understanding of the extent to which oxidative stress is a part of the disease. A second explanation is that the type and dosage of antioxidants used in the clinical trials may not have attenuated oxidative stress in a tissue- or cell-specific fashion and therefore had no effect or caused harmful effects. This explanation can be taken into account since the antioxidant system is complex and interconnected. For instance, SOD enzymes catalyze the disproportionation of O_2_•− and subsequently generate another ROS such as H_2_O_2_, as a product (Figure 3); for example, the antioxidant vitamin E acts on the level of a lipid membrane, whereas vitamin C acts in the extracellular and intracellular aqueous milieu; and both higher and lower ingestions of vitamin C, compared with recommended daily dose, are associated with oxidative DNA damage (Nikolić et al., 2006). Moreover, the reason that clinical trial studies have generally failed to confirm benefits of antioxidants may be that the antioxidants can generate harmful effects in some cases. For example, the dual action (anti- and prooxidant) of vitamin C was confirmed by many molecular studies (Zahra et al., 2021). The pro-oxidant effects of antioxidants are relevant in the pathology and management of cancer, when sufficient amount of ROS is needed to induce apoptosis of malignant cells (Son et al., 2015). Additionally, some antioxidant therapies fail due to the undesirable disruption of physiological functions caused by ROS, since ROS are not only by products of metabolic reactions but are also involved in physiological signaling and thus the alteration of the redox homeostasis promotes various metabolic outcomes by mediating various metabolic processes. Finally, clinical trials in humans come out inconclusive due to the lack of a suitable method for quantifying redox status, or the redox status was not assessed before and after initiation of antioxidant therapy in many clinical trials (Murphy et al., 2011). In addition, measuring one or several pro- and antioxidant markers may not be reflective of a complete measure of redox status of the target organ or tissue, and the systemic redox status may not be characteristic of the status of the target.

## 6 Conclusion

Chronic inflammation is often associated with increased oxidative stress, where the balance between the production of ROS and the body’s ability to detoxify them is disrupted. ROS can damage cellular components such as DNA, proteins and lipids, contributing to the progression of inflammatory diseases. Neutralizing ROS, various antioxidant substances (natural and synthetic) may help to reduce oxidative stress and limit tissue damage in chronic inflammatory conditions. Regarding natural antioxidants, source and dosage deeply impact the effects seen, with bioavailability apparently a major factor in obtaining the desired outcomes. Despite the fact that natural antioxidants are considered safer than synthetic ones, their concentration in medicines is significantly higher than in raw organic sources (fruit and vegetables), requiring a detailed investigation of potential side effects (Salehi et al., 2018). Further randomized human clinical studies are required to determine the optimal therapeutic dose of drugs derived from natural antioxidants to evaluate their risks and benefits. Notably, direct binding of ROS inside the cell results in lower antioxidant effect compared to the activity of antioxidant enzymes, for example, SOD, which has a reaction rate constant 105 times greater than that of most antioxidants in the reaction with O_2_•−. That makes the contribution of exogenous antioxidant compounds negligible compared to the activity of endogenous antioxidant enzymes (Forman and Zhang, 2021). A therapeutic strategy aimed at regulating the activity or expression of antioxidant enzymes and enzymes involved in the production of ROS seems to be more effective. In this regard, it is advisable to search for new natural compounds that can participate in the regulation of the cellular antioxidant enzymatic system.

As for synthetic antioxidants, we have described a number of promising drug candidates, which are mimetics of antioxidant enzymes, inhibitors of the mitochondrial ROS formation, NADPH oxidase inhibitors, as well as activators of the Nrf2 pathway. We hope that all these approaches will promote the further development of antioxidant therapy for chronic inflammatory diseases. Additional studies are necessary to carefully assess the systemic side effects and carefully select the dose of novel drugs.

## Figures and Tables

**Table:** 

**AJs**	Adhesion proteins
**Akt**	Protein kinase B
**AP-1**	Activator protein 1
**Apaf-1**	Apoptotic peptidase activating factor 1
**ATP**	Adenosine triphosphate
**Bcl-2**	B-cell lymphoma 2 gene
**CAT**	Catalase
**CHF**	Congestive heart failure
**CVDs**	Cardiovascular diseases
**DAMPs**	Damage-related molecular patterns
**DNA**	Deoxyribonucleic acid
**Erk**	Extracellular signal-regulated kinase
**EUK-34**	Manganese -3-methoxy N, N′-bis (salicylidene) ethylenediamine chloride
**EUK-8**	Manganese (salen) chloride
**GPx**	glutathione peroxidase
**Grx**	Glutaredoxins
**H2O2**	Hydrogen peroxide
**HOCL**	Hypochlorous acid
**IFNγ**	Interferon gamma
**iNOS**	Inducible nitric oxide synthase
**Keap1**	Kelch-like ECH-associated protein 1
**MAPK**	mitogen-activated protein kinase
**MnP**	Manganese porphyrin
**MnSOD**	manganese SOD
**MnTDE-2-ImP5+**	Manganese(III) meso-tetrakis (di-N-ethylimidazole) porphyrin
**MnTE-2-pYp5+**	Manganese(III) meso-tetrakis(N-ethylpyridinium-2-yl)porphyrin
**MnTM2-pYp5+**	Manganese (III) meso-tetrakis (N- methylpyridinium-2-yl)porphyrin
**NADPH**	Nicotinamide adenine dinucleotide phosphate
**NADPH**	nicotinamide adenine dinucleotide phosphate
**NF-kB**	Nuclear factor kappa-light-chain-enhancer of activated B cells
**NOS**	Nitric oxide synthase
**Nrf2**	Transcription factor
**O2•−**	Superoxide anion
**OH−**	Hydroxyl anion
**•OH**	Hydroxyl radical
**ONOO-**	Peroxynitrite
**PI3K**	Phosphoinositide-3-kinase
**PPARγ**	Peroxisome proliferator-activated receptor gamma
**Prx**	Peroxyredoxins
**ROS**	Reactive oxygen species
**SOD**	Superoxide dismutase
**TEMPOL**	4-Hydroxy-TEMPO
**TJs**	Tight junction proteins
**TNFα**	Tumor necrosis factor alpha
